# Vertical split fracture of the vertebral body following oblique lumbar interbody fusion: A case report

**DOI:** 10.1097/MD.0000000000029423

**Published:** 2022-05-27

**Authors:** Jong-Hwan Hong, Moon-Soo Han, Jung-Kil Lee, Bong Ju Moon

**Affiliations:** a Department of Neurosurgery, Chonnam National University Hospital, Gwangju, Republic of Korea; b Department of Neurosurgery, Chonnam National University Hospital and Medical School, Gwangju, Republic of Korea.

**Keywords:** anterior column release, oblique lumbar interbody fusion, osteoporosis, vertebral fracture

## Abstract

**Rationale:**

Oblique lumbar interbody fusion (OLIF) is an effective and safe surgical technique widely used for treating spondylolisthesis; however, its use is controversial because of several associated complications, including endplate injury. We report a rare vertebral body fracture following OLIF in a patient with poor bone quality.

**Patient concerns:**

A 72-year-old male patient visited our clinic for 2 years with lower back pain, leg radiating pain, and intermittent neurogenic claudication.

**Diagnoses:**

Lumbar magnetic resonance imaging revealed L4-5 stenosis.

**Intervention:**

We performed OLIF with percutaneous pedicle screw fixation and L4 subtotal decompressive laminectomy. We resected the anterior longitudinal ligament partially for anterior column release and inserted a huge cage to maximize segmental lordosis. No complications during and after the operation were observed. Further, the radiating pain and back pain improved, and the patient was discharged. Two weeks after the operation, the patient visited the outpatient department complaining of sudden recurred pain, which occurred while going to the bathroom. Radiography and computed tomography revealed a split fracture of the L5 body and an anterior cage displacement. In revision of OLIF, we removed the dislocated cage and filled the bone cement between the anterior longitudinal ligament and empty disc space. Further, we performed posterior lumbar interbody fusion L4-5, and the screw was extended to S1.

**Outcomes:**

After the second surgery, back pain and radiating pain in the left leg improved, and he was discharged without complications.

**Lesson:**

In this case, owing to insufficient intervertebral space during L4-5 OLIF, a huge cage was used to achieve sufficient segmental lordosis after anterior column release, but a vertebral body coronal fracture occurred. In patients with poor bone quality and less flexibility, a huge cage and over-distraction could cause a vertebral fracture; hence, selecting an appropriate cage or considering a posterior approach is recommended.

## 1 Introduction

Oblique lumbar interbody fusion (OLIF) is an effective and safe surgical technique widely used for treating lumbar degenerative diseases with spondylolisthesis. This approach can preserve the posterior component of the spinal column compared to the posterior approach. Recent studies have shown that OLIF is a low-morbidity, reliable, and effective method for treating degenerative lumbar stenosis and spondylolisthesis owing to rapid recovery and early ambulation after surgery.^[1,2]^

However, despite many advantages, OLIF cannot directly decompress the nerve root and is controversial because of several associated complications, such as muscle and blood vessel injury, lumbar plexus injury, and urethral injury.^[3,4]^ In particular, Walker et al has reported that the incidence of endplate damage and subsequent cage subsidence can reach up to 15%, leading to a poor prognosis.^[3,5]^ However, a few studies have reported vertebral body fractures after OLIF surgery. We report here a vertebral body fracture after OLIF in a patient with poor bone quality.

## 2 Case presentation

The patient has provided informed consent for the publication of the case.

**Figure 1:**
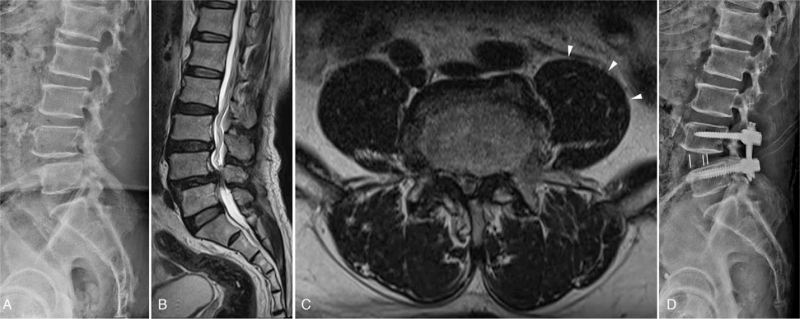
Perioperative imaging for the first operation. (A) Lateral radiographs of the lumbar spine demonstrate L4-5 grade I spondylolisthesis. (B) Lumbar MRI indicates L4-5 stenosis. (C) Preoperative lumbar MRI indicates enlarged left iliopsoas muscle (white arrows). (D) Postoperative radiography shows a relatively anterior location of the cage. MRI = magnetic resonance imaging.

A 72-year-old male patient visited our clinic for 2 years with lower back pain and lateral to posterior leg radiating pain. His physical examination did not reveal motor weakness on initial presentation, but he complained of intermittent neurogenic claudication of fewer than 50 m. Lumbar magnetic resonance imaging showed L4-5 stenosis (Fig. 1). Lateral radiographs of the lumbar spine demonstrated L4-5 grade I spondylolisthesis without significant instability (Fig. 1). The patient was on dialysis for end-stage renal disease and was accompanied by hypertension and diabetes. The T-score of the bone mineral density (BMD) of the femur neck was −1.5, and the lumbar BMD was within the normal range. The authors decided on OLIF with percutaneous pedicle screw fixation and L4 subtotal decompressive laminectomy.

The oblique anterior-to-psoas approach was first performed at the right lateral decubitus position. Considering enlarged iliopsoas muscle on preoperative lumbar magnetic resonance imaging, the operator approached more anteriorly and performed sufficient discectomy without endplate damage (Fig. 1). To maximize segmental lordosis, we resected the anterior longitudinal ligament partially for anterior column release (ACR) and attempted to insert a large cage, which could be pulled out repeatedly. The cage (Cougar LS lateral cage system, DePuy Synthes, USA) with a depth of 18 mm, 15° was inserted. After changing to the prone position, percutaneous bilateral pedicle screw fixation and bilateral L4 total laminectomy were performed. Postoperative radiography showed a relatively anterior location of the cage (Fig. 1). No complications during and after the operation were noted. Further, the radiating pain and back pain improved and the patient was discharged.

**Figure 2:**
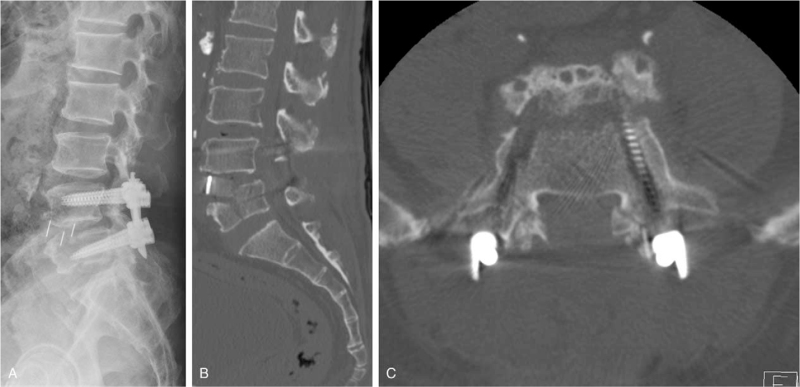
Preoperative imaging for revision operation following sudden back pain and radiating pain in the left leg. (A–C) Radiography and computed tomography show a split coronal fracture of the L5 body and an anterior cage displacement.

Two weeks after the operation, the patient visited the outpatient department complaining of sudden back and radiating pain in the left leg, which occurred while going to the bathroom. The radiography and computed tomography (CT) revealed a split fracture of the L5 body and an anterior cage displacement; hence, we decided to reoperate the patient (Fig. 2).

In revision of OLIF, the dislocated cage was removed, and the anterolateral screw fixation was attempted; however, the screws could not be tightly inserted owing to poor bone quality and fracture. We filled the anterior longitudinal ligament and empty disc space with bone cement (Spinofill, INJECTA, Republic of Korea). After changing the prone position, we performed posterior lumbar interbody fusion L4-5, and the screw was extended to S1. The cages were inserted, and they were in contact with the bone cement on both sides. After surgery, back pain and radiating pain in the left leg improved, and he was discharged without complications.

**Figure 3:**
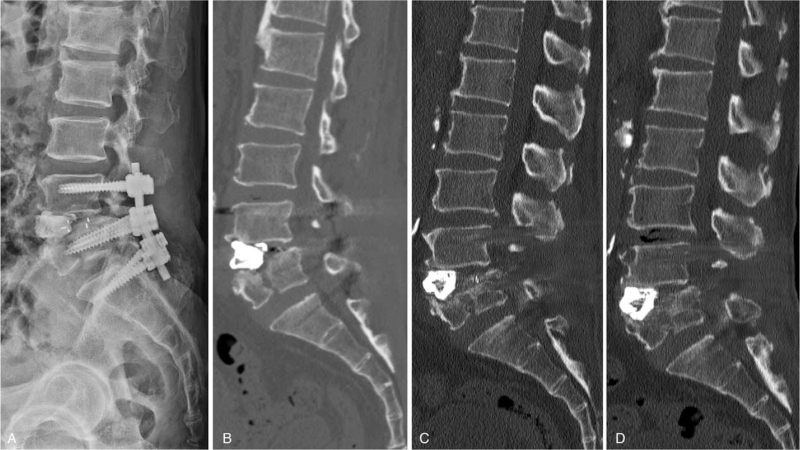
Serial follow-up postoperative imaging. (A) Postoperative lateral lumbar radiography showed posterior lumbar interbody fusion L4-5 and the screw extended to S1 with interbody cement augmentation. (B) CT after 6-months follow-up showing that the bone fragment is not fused. (C) CT after a 1-year follow-up showing an interbody space and bone fragment fused partially. (D) CT after a 2-years follow-up showing interbody space and bone fragment fused solid. CT = computed tomography.

Teriparatide, as an outpatient treatment, was administered for clinical osteoporosis due to bony fusion. At 1 year of follow-up, CT revealed that the bone union was occurring (Fig. 3). Two years after the second operation, CT displayed L4-5 body solid fusion along with the patient's favorable condition (Fig. 3).

## 3 Discussion

OLIF is a minimally invasive surgery that allows lumbar interbody fusion. Compared to other direct decompression procedures such as posterior lumbar interbody fusion, OLIF can reduce intraoperative bleeding, preserve the paravertebral muscles and spinal column, and improve postoperative pain and early recovery.^[2,6]^

Despite many advantages, surgical corridor-associated complications, such as vascular injury, lumbar plexus injury, sympathetic chain injury, vertebral endplate fractures, and postoperative thigh sensorimotor impairment, can occur.^[4,7,8]^ Cage subsidence or movement due to endplate injury during surgery is considered a common postoperative complication, and minimizing endplate damage in the course of the disc space preparation is an essential technique for the OLIF prognosis. Vertebral fractures during OLIF surgery are rare and usually the result of endplate injury.^[9–11]^

However, similar to this case, vertebral fractures after OLIF without endplate injury have been barely reported. Some authors have reported vertebral fractures after extreme lateral interbody fusion or lateral lumbar interbody fusion applied large cages, but these were coronal plane fractures related to lateral screw fixation.^[12,13]^ We discuss here the circumstance of this case through a literature review of OLIF.

In cage selection, Zhang et al showed that endplate collapse is more likely to occur with short half-span cages.^[14,15]^ In our case, the width of L5 reached 55 mm, but a cage of 45 mm was applied. A vertebral fracture might have occurred because the cage did not sufficiently span both ends of the epiphyseal ring and was located on the central concave side of the endplate in the lower vertebra, which is relatively weak compared to the ring. Therefore, choosing a long enough cage from both sides to support it on a more rigid epiphyseal ring might reduce the risk of lower vertebral body fractures.

The appropriate anteroposterior position of the cage depends on the operator's preference, and some authors have shown that the anterior location of the cage could provide a higher sagittal angle and foramen height.^[16,17]^ In addition, some authors have reported that ACR can create more significant segmental lordosis.^[18,19]^ In this case, the absence of enough intervertebral space even after discectomy and huge cage placed anteriorly by ACR effectively resulted in segmental lordosis, ranging from 7° preoperatively to 26° after surgery. In contrast, the intervertebral height did not significantly increase from 8 mm to 10 mm, probably owing to decreased flexibility of the posterior element. After ACR, under the decreased anterior contraction and still rigid posterior element, over-distraction owing to the vast cage might result in a greater concentration of forces on the posterior area of the cage, which could lead to coronal fractures in the vertebral body beyond the endplate damage. This is consistent with a report by Shiga et al describing a relatively anterior cage that increases the risk of endplate damage.^[20]^ For difficulty to place a high and large angle cage rather than to try a high-angle cage, more extended cage depth can increase the cage area and reduce the pressure on the bony interface, thereby lowering the risk of fractures to the lower vertebrae. A posterior approach, instead of OLIF, is a good alternative if it is difficult to place a sufficient-sized cage due to decreased flexibility on the dynamic study before surgery.

Given the underlying disease, the BMD was within normal range, but the patient presented chronic kidney disease and poor bone quality during surgery. Osteoporosis is a poor prognostic factor for lumbar arthrodesis, which increases the risk of nonunion.^[21]^ In this case, poor bone quality might have increased the risk of fracture. Attempts to improve the prognosis of lumbar interbody fusion in osteoporotic patients continue, and the use of teriparatide has increased recently. Yolcu et al have showed that using teriparatide before lumbar interbody fusion can reduce postoperative osteoporosis-related complications.^[22]^ In our case, after the second operation, teriparatide was continuously administered, and bone fusion was achieved 2 years after the operation.

## 4 Conclusion

ACR and a large anterior cage are useful to achieve sufficient segmental lordosis. In this case, because of insufficient intervertebral space during L4-5 OLIF, a huge cage was used to achieve sufficient segmental lordosis after ACR, leading to vertebral body coronal fracture. In patients with poor bone quality and less flexibility, a huge cage and over-distraction could cause a vertebral fracture; hence, selecting an appropriate cage or considering a posterior approach is recommended.

## Author contributions

**Investigation:** Bong Ju Moon.

**Supervision:** Bong Ju Moon.

**Validation:** Bong Ju Moon.
